# Adaptation of a community health outreach model during the COVID-19 pandemic: the case of the Mexican consulates in the United States of America

**DOI:** 10.1186/s12939-023-01911-9

**Published:** 2023-07-25

**Authors:** Pablo Gaitán-Rossi, Mireya Vilar-Compte, Arturo Vargas Bustamante

**Affiliations:** 1 Research Center for Equitable Development EQUIDE, Universidad Iberoamericana Mexico City, Mexico City, Mexico; 2 Department of Public Health, Montclair State University, Montclair, NJ USA; 3 Department of Health Policy and Management, University of California Los Angeles, Los Angeles, CA USA

**Keywords:** Access to health care, COVID-19 pandemic, Hispanic or Latino health, Immigrant health, Crises preparedness

## Abstract

The Ventanillas de Salud (VDS – “Health Windows”) are a culturally sensitive outreach program within the 49 Mexican Consulates in the United States that provides information and health care navigation support to underserved and uninsured Mexican immigrants. During the COVID-19 pandemic the VDS rapidly transitioned to remote operations adding new services. Based on the EquIR implementation framework, this qualitative study investigates how adaptations to improve emergency preparedness were performed. We conducted motivational interviews with three actors – six VDS coordinators, eight partner organizations, and ten VDS users– in two VDS, Los Angeles and New York, to document specific needs of the target population and identify implementation processes to adapt and continue operating. The VDS adapted their model by adding new services for emerging needs, by switching service provision modalities, and by expanding the network of partner organizations. According to the VDS staff, these adaptations increased their topics, depth, reach, and diversified their users. Users had mostly positive opinions about the VDS adaptation, although they highlighted some heterogeneity across service provision. The VDS is a public health intervention able to serve a marginalized population and its implementation offers valuable lessons to complement health systems and to improve preparedness and resiliency for future crises.

**Supplementary Information:**

The online version contains supplementary material available at 10.1186/s12939-023-01911-9.

## Introduction

Analyses of COVID-19 transmission dynamics in the United States (US) revealed that the risk of infection and death varied amongst populations, and social vulnerability was a key predictor of important health disparities [1]. The US Hispanic population had the largest decline in life expectancy (3 years) between 2019 and 2020 compared to other race/ethnicities, mostly because of COVID-19 [2]. Poor Hispanic communities had an increased risk of COVID-19 infection– especially communities that concentrated monolingual Spanish speakers [3]. In California, working-age Hispanics had the highest relative excess mortality (37%) during the first nine months of the pandemic – with the highest excess mortality among Hispanic workers in the food and agricultural sectors (59%) [4].

Several underlying factors explain the disproportionate COVID-19 burden on Hispanic populations. First, Hispanics have higher rates of known COVID-19 risk factors than non- Hispanic whites, such as obesity, hypertension and diabetes [5, 6]. Second, racial/ethnic minorities and low-income individuals living in urban areas are less likely to have a constant monthly income and remote jobs with digital access [7]. In addition, Hispanic migrants have lower insurance coverage and access to health care than the US-born population [5]. Some of the barriers that hinder health-care access are low English proficiency, non-citizenship status, fears of deportation, lower contextual based perceptions of trust and safety [8], and discrimination [9]. These underlying factors and the COVID-19 morbidity and mortality disparities suggest that Hispanic populations have specific needs and that commonplace prevention efforts, such as shelter-in-place, are less effective among them [7].

Amongst Mexicans in the US, an important strategy to get information and health care navigation support during the COVID-19 pandemic was seeking help from the Mexican Consulates. Particularly, the Ventanillas de Salud (VDS) have been found to be a culturally sensitive outreach program within the Consulates available to Mexicans in the US regardless of their immigration status [10]. Starting in 2003 as a joint initiative between the Mexican Ministries of Health and Foreign Affairs, the goal of the VDS has been to provide health information and facilitate access to health care and local community resources for Mexicans living in the US [11]. Even though the Mexican government funds the VDS, they partner with different public and private organizations and coordinate actions with federal, state, and local US governments. The VDS provide free, culturally, and linguistically sensitive basic health services in a safe environment. A recent scoping review found that VDS mostly offer three types of services: (1) healthy lifestyles information and counseling; (2) immunizations and early disease detection; and (3) referral to community clinics [10]. The VDS are located within 49 Mexican Consulates in the US with mobile clinics that complement their services. VDS serve an estimated 1.5 million individuals per year [12].

The COVID-19 pandemic disrupted health systems worldwide including basic services unrelated with the pandemic [13, 14]. Hospital conversion was a challenge for health facilities aiming to provide health care in safe conditions [15]. VDS are well-positioned to improve health outcomes by reducing health misinformation and providing basic preventive screenings and health care navigation support from an equity and people-centered perspective. Moreover, the VDS partner with akin organizations to expand their services, outreach and community networks to hard-to-reach population [16]. In addition, the VDS community engagement contributes to establishing trust between underserved and uninsured Mexican immigrants and the US health system, hence, they can have a pivotal role in contributing to a diverse and resilient health system by integrating a systematically excluded population [17]. For example, COVID-19 vaccination can be a case in point for the policy relevance of the VDS. The June 2021 Kaiser Family Foundation Vaccine Monitoring reported the stalling rates of vaccination in the US. It highlighted that two groups served by VDS, non-insured, and Hispanic adults, are some of the groups that will rather “wait and see” before getting vaccinated [18]. Importantly, Hispanic adults recognize that mobile clinics are amongst the top interventions that might motivate them to get the vaccine [18]. This is the type of outreach effort where the VDS might excel from other health care programs because they can benefit from being a trusted and culturally sensitive source. Previous research has shown that VDS rapidly transitioned to remote operation such as providing health information and health care navigation support to Hispanic immigrants during the COVID-19 pandemic [16]. However, to our knowledge, no prior research has investigated how the adaptations to remote care during the COVID-19 pandemic were implemented, which is a relevant question to improve crises preparedness.

The objective of the study is to examine how the VDS in two Mexican Consulates in the US adapted their outreach services to better serve Mexicans in the US during the COVID-19 pandemic. By targeting a marginalized population that has been systemically excluded from several types of health and social services, it is important to examine these adaptations from an equity perspective. Therefore, we use the EquIR framework [19], a pragmatic guideline for conducting equity-focused implementation research in health interventions. We identify the key implementation processes that the VDS enacted to adapt and continue outreach services and document the specific needs and experiences of the population that used the VDS to cope with the COVID-19 pandemic. The qualitative study will assess implementation outcomes that aid in the strengthening and replication of the VDS model for future public health crises.

## Methods

Following an implementation science approach, we used a qualitative process evaluation design focusing on the case of the VDS as to describe an intervention during a specific context to understand the complexities affecting adoption and sustainability [20, 21]. The study was conducted in the Mexican Consulates in New York City (NYC) and Los Angeles, California (LA), in the US. Nearly half of the US immigrant population in 2017 was comprised by immigrants from Latin America [22] including Mexico, which remains as the country with the largest number of migrants living in the US (over 11.9 million, accounting for 25% of all US immigrants) [23]. LA and NYC have a large share of Hispanics. According to the 2019 Census Bureau [24], the national share of the Hispanic population was 18.5%, while in LA and NYC it was 48.6% and 29.1%, respectively [25]. In spite of being both “sanctuary cities”, they differ in migration patterns and acculturation; with LA being a long-standing enclave for Hispanics and NYC with a more recent history of migration following work-related patterns, with localized networks, as from the state of Puebla [26, 27]. There are considerable variations amongst the VDS in the US, especially in size and services [10], so we chose two of the largest VDS, embedded in sanctuary cities with a rich network of social organizations helping Hispanics. The selection allows us to assess robust VDS and observe its current potential to become models for other VDS and similar outreach organizations.

The study is based on online one-hour semi-structured motivational interviews with three types of key actors for the adaptation of the VDS model: VDS coordinators (N = 6), key informants of partner organizations (N = 8), and VDS users (N = 10). Motivational interviews are an interview style that involves identifying participant values that support a change in behavior, in this case, in decision-making around the adaptation of services, and the interaction with the adapted services. The interview process requires a reflection to understand if cognitive dissonance exists, which would imply that participants’ actions around adaptations do not reflect their stated values [28].

We used a two-pronged snowball sampling approach. First, we interviewed the VDS coordinators at two-points in time: in October 2020 –eighth months from the onset of the pandemic–, and twice in June 2022 –when large portions of the population were vaccinated, and activities were returning to normality. The first set of interviews helped understand the first adaptations to the pandemic and the second set offered insights on specific issues of its later stages, such as vaccinations. Then, in 2022, the VDS coordinator in each Consulate provided contact information of key informants in partner organizations that worked with the VDS to serve the Hispanic population during the pandemic. Researchers then invited all the managers or key staff from such partner organizations to participate in the study. We were able to contact 4 out of 6 recommended organizations in NYC and 4 out 6 in LA; 3 organizations did not respond to our emails and with another we were unable to schedule an interview. The interviews with the partner organizations were conducted between June and August 2022.

The VDS coordinators invited users to participate in the study. The VDS coordinator in LA sent an email to the registered VDS users during the pandemic period with an invitation to contact the principal investigator in Mexico. As an incentive, we offered a $30 USD gift card to those who matched the inclusion criteria (i.e., used the VDS during the pandemic and agreed to a one-hour interview). After receiving the email, users expressed interest within the next five days. Interviews with VDS users took place between July and August 2022. The VDS in NYC contacted VDS users posting the same flyer in their Facebook website, a popular site to communicate with VDS users and disseminate information during the pandemic. However, nobody contacted the research team. This is likely resulting from the indirect recruitment strategy implemented NYC.

## Measures

The interviews targeted the adaptation of the organizations from the perspectives of each of the three actors; see supplementary material 1 for all questions in each guide. For the interview protocol, we first asked about the successes, implementation challenges, and unattended issues to rapidly transform the VDS or partner organizations at the onset and progression of the COVID-19 pandemic. We emphasized collaboration processes and descriptions of the changing needs and services provided during the different phases of the pandemic (i.e., COVID-19 information, diagnostic testing, government support, medical and psychological care, and vaccination). The topics of the interview with VDS users were their health and economic needs during the pandemic, the reasons and expectations in using the VDS, their experience with the VDS, the combination of health care services and providers, the satisfaction with the VDS, and recommendations to improve its services.

The one-hour interviews with the VDS coordinators and the key informants from the partner organizations were conducted in Spanish by the Co-PI in Mexico (except for one, in English), and the one-hour interviews of the VDS users were also conducted in Spanish by a trained research assistant with previous experience in qualitative research. All interviews were recorded and took place in the ZOOM digital platform, most of the time with video. Interviews were transcribed with the SONIX software and then unidentified prior to the analyses.

## Analysis

For our analytic approach we used an equity framework based on implementation science – the EquIR [19]. Implementation aims to understand “how” and “why” implementation efforts succeed or fail [29]. The EquIR framework was developed to “incorporate equity issues during the whole process of planning, designing, implementing, and monitoring the health program or intervention” [19]. It focuses on four components: context and needs, strategies for innovation, implementation outcomes, and equity outcomes. The EquIR framework aims to evaluate seven implementation outcomes – acceptability, adoption, appropriateness, feasibility, fidelity, implementation cost, coverage, and sustainability [19] – which are defined in Table 1. The EquIR framework helps understand how the VDS transformed its services due to the pandemic and how they can be better prepared for the next public health crisis.

**Table 1:** Categories used for thematic analyses, based on the EquIR framework

Nodes	Sub-nodes	Definitions
**Context and needs**	Health status	Perceived and diagnosed health conditions of the target population of the VDS and partner organizations – Hispanic and Mexican immigrant population in the US during the pandemic.
	Health inequities	Perceived health differences and specific health need in the target population.
	Stakeholders	Organizations (public or private) contributing to the implementation of the VDS.
**Strategies for adaptation and innovation**	Services	Continuation of regular services and service innovations due to the pandemic.
	Communication strategies	Processes for the delivery and reception of health information with an equity approach.
	Barriers and facilitators	Situations that prevent or favor service implementation.
	Recommendations	Aspects for improvement to overcome the barriers identified and continue with the implementation with an equity approach.
**Implementation outcomes**	Acceptability	Perception of stakeholders in implementation.
	Adoption	The intention or utilization of equity elements into implementation.
	Appropriateness	Relevance or perceived fit of the implementation in the disadvantaged population.
	Feasibility	Extent to which a program can be carried out in any setting, especially among disadvantaged populations.
	Fidelity	Adherence of disadvantaged population to the intervention.
	Implementation cost	Costs involved in the implementation of equity-focused elements.
	Coverage	Degree of reach, access, and coverage of the intervention among the disadvantaged population.
	Sustainability	Maintenance, continuation, or durability of the program through short, medium, and long-term strategies.

The thematic analyses followed a concept coding method [30] based on the EquIR framework. A research assistant and one of the principal investigators independently coded the materials in the original language they were collected (as all researchers are bilingual), following a codebook stemming from the EquIR framework (see Table 1 for details about nodes and sub-nodes) and with a line-by-line approach. Discrepancies were handled first between the two coders. On two occasions that discrepancies persisted a third researcher gave her input to resolve the issues. The coding process began with the experiences of the participants elicited by the interview protocol, then these experiences went to higher level sub-nodes and nodes following the predetermined structure of the EquIR framework. The first three parts of the results section reflect the findings from the components context and needs and strategies for innovation. The fourth part focuses on the implementation outcomes but given space constraints, a more detailed account of selected quotes for implementation outcomes by city and by type of informant can be found in supplementary material 2. Analyses were done in DEDOOSE, version 8.3.47, a software for managing, analyzing, and presenting qualitative research.

The study was approved by the Research Ethics Committee of the Universidad Iberoamericana (Reg. No. CONBIOETICA-09-CEI-008-2016060). A written informed consent was sent prior to the interview for all three groups, once the participants showed interest in participating in the study. After answering all their questions about the study, an oral informed consent was requested before beginning the recorded interview. We purposefully avoided any questions on migratory status.

## Results

### Service provision during the COVID-19 pandemic

The VDS had to adapt their services at the onset of the pandemic. As informants from the VDS expressed, they changed the focus of their standard services from offering information on the health system and prevention of chronic diseases, to attend the “most urgent needs of the pandemic” and strengthen “food and financial security.” As described by one of the VDS informants, “we became an organization for humanitarian support”. This meant the VDS had to provide new information and referrals, such as the location to get COVID-19 tests, vaccines, and medical services, as well as specific demands of their population, like the procedure to repatriate a corpse. The VDS coordinators explained they also designed informative courses and workshops to counter COVID-19 misinformation, provide reliable advice, promote wellbeing, and improve digital literacy (i.e., illustrating how to get an online appointment to get a vaccine). The VDS coordinators added that they shifted their operations to an online modality, and expanded their health and social care services, such as initiating remote mental health support, and information support on how to access social services, particularly unemployment support, rent benefits, and food banks.

The VDS services evolved during the pandemic. The VDS coordinators mentioned how they started by just providing online information on relevant COVID-19 topics and then added direct services depending on the stage of the pandemic. Based on their accounts, some of the new prominent services included: food pantries and economic benefits to reduce food insecurity; disbursement of medicines for chronic disease management; and delivery of COVID-19 personal protection equipment (i.e., masks), testing, and vaccinations. According to the VDS staff, the shift in priorities meant that they interrupted or reduced their standard service provision and chronic disease screening and management were the standard services most affected.

The VDS coordinators considered they had limited staff during the pandemic, increased workload, and high turnover, which translated into self-reported burnout and mental health issues. For instance, one VDS started the pandemic with one person, then increased to three, but went back to two persons. In their opinion, staff rotation interrupted communication and the earned trust with partner organizations, limiting outreach efforts. As a VDS coordinator reflected: “We couldn´t provide our regular services to the community, we had to figure out how to adapt (…), and the greatest difficulty was that it happened suddenly, you had to move on, but with additional workload. And those difficulties relate with mental health issues”.

The VDS coordinators recalled that, at the onset of the pandemic, the Consulates closed for two months, and everyone worked remotely and relied on one-to-one virtual calls. The VDS staff preferred ZOOM to deliver their services. They communicated their services through social media (Facebook and Twitter), emails, and their website; with nearly 4,000 followers in social media per Consulate. Interestingly, some users mentioned they received services from the closest VDS but often they preferred to read information from a different VDS, suggesting that the communication reach is usually wider than their actual area of service. According to the VDS coordinators, by October 2020, as lockdown measures were relaxed, some services resumed, and health fairs and community visits reopened. At this stage of the pandemic, they handed out flyers with information of important events and occasionally publicized their services in local radio stations. The VDS staff expressed that, throughout the pandemic, word of mouth was the most effective strategy to disseminate information among their target population because users were key to promote their services and replicate information.

### Network of organizations

The VDS are embedded in a large network of three main types or organizations: institutions providing a service, organizations linked to the community without providing a direct service, and public organizations at different levels. The closest VDS partners were non-for-profit organizations offering health and social services in diverse sectors, such as health care, education, homelessness, alcoholism, and legal advice. To avoid fear of documentation status or other migratory ethnicity-based biases, these organizations do not ask for identification when offering their services, as one partner underscores, “the work has always been about reaching those that are hard to reach”. These organizations share with the VDS a concern to reduce access barriers to health and social services; a member of one of these partner organizations explained that, regardless of the immigration or insurance status of their users, “we will make sure no one leaves the clinic without health care, either low-cost or free, and with a program they are eligible to receive.” A special case were temporary non-for-profit organizations created during the pandemic to offer ad hoc services (i.e., food pantries), not necessarily tailored for Hispanics but tapping on their pressing needs.

Based on the interviews and examples provided by the VDS, a second type of partner organizations were those not providing direct services, like Churches and other Consulates (especially from Central American countries such as Guatemala, El Salvador and Honduras) but which helped promote the VDS and disseminated relevant information. In addition, important stakeholders were federal and state government agencies (i.e., mental health offices and children’s hospitals) that supported the VDS and other local organizations to provide preventive and primary health care to this population.

This network of organizations consolidated and expanded during the pandemic. Both the VDS and the partner organizations recognize that previous partnerships were instrumental for all organizations, including the VDS, to implement new services. First contacts between organizations were initiated by the VDS or by the organizations that saw strategic advantages in the Consulate to reach their target populations. Likewise, each organization went through an adaptation process to better respond to pandemic needs. Former personal relationships were key for these adaptations because staff members already trusted the partner’s previous services, communicated often, and some characteristics of these organizations were important for the provision of the new services. For instance, VDS and its community partners continued to offer services in Spanish, at no cost on the point of service, and practitioners had cultural competency and encourage utilization despite of user’s migratory status. These networks got stronger and more reliable during the pandemic because they increased the frequency of their communications, and they implemented additional case follow-up to ensure VDS users received service after referrals. The next account by a VDS reflects how networks organized for shared goals:

When the vaccine was about to come out, we [VDS] started mobilizing to see who we might partner with to offer this service. (…) We were able to contact the Department of Public Health. The relationship already existed, but the Department has several branches, and by asking, little by little, we created the relationship. By the end of January 2022, we found the right department, who invited us to join their mobile clinics. (…) At first, they asked us to join efforts with one of our partners, one of our oldest strategic allies. That´s how we started with our vaccination rallies, we made 4 in total, administering 500 Covid vaccines. Later on, chances were more open and another one of our important partners called us to suggest a new collaboration with the City and it was until then that we are able to offer the vaccine Monday thru Saturday, in addition to the tests we were already giving. It was a perfect fit because that way everyone could just come to us [at the Consulate].

### Users of health and social services

The VDS and partner organizations recognize that their users are an important element in this network of organizations because they help identify needs, offer feedback on the services, and promote the new services on their own interpersonal networks. Both types of organizations mentioned examples of how they outreach to similar vulnerable populations: low-income, Spanish-speaking Hispanic populations (mostly from Mexican origin), living in large households, oftentimes uninsured, without legal documentation, and “segregated in neighborhoods with high concentrations” of Hispanics – in the words of a partner organization. As one VDS user expressed: “We live in this country with fear to many things, like the police and migration officers”.

During the pandemic, informants from partner organization explained that many VDS’s users were unemployed or providing essential services, among the latter, “they never stopped working, cleaning, at restaurants, they were cashiers, and doing domestic work.” Another informant from a partner organization confirmed that “a lot of the patients had these kinds of jobs where they were exposed to a lot of people and therefore getting COVID at a higher rate.” On the importance of providing sensitive and trust-based services during the pandemic, one organization stated that “it was evident at the beginning they did not want to take the vaccine because they thought it would damage their future migratory status.” This fear is consistent with “public charge” concerns. Hence, the VDS personnel acknowledged that the target population has specific needs that grant an equity approach to service provision.

As summarized in Table 2, users in our sample were 47 years old on average, 9 graduated from high school, half reported having been diagnosed with a chronic disease, and about one quarter (27%) were uninsured. The VDS users in our sample can be broadly divided into two groups. The first group comprises the regular users for whom the services were designed. They have Mexican origins and mostly sought information on COVID-19 and advice on how to navigate the health system. Besides COVID-19, they also sought healthcare related with chronic diseases, alcoholism, and diverse mental health issues, particularly depression.

**Table 2:** Characteristics of VDS users (N = 11)

ID	Education level	Age	Chronic disease	Health insurance
1	Middle school	53	Diabetes	Yes
2	Middle school	47	No	No
3	High School	44	No	No
4	High School	71	Hypertension	Yes
5	High School	45	No	Yes
6	High School	56	Diabetes and Hypertension	Yes
7	High School	37	Diabetes	Yes
8	High School	40	No	No
9	Master’s degree	41	No	Yes
10	Bachelor’s degree	42	Arthritis	Yes
11	High School	NA	NA	Yes

The second group of VDS users includes community health workers working for several health and social services organizations (sometimes without a salary). The key advantage of these community health workers (not always from Mexican origin) is that they are already embedded in Hispanic networks and are trusted by the community. According to the VDS personnel, since some lack a formal education, they frequently take the Consulate’s courses and workshops to stay updated and increase their knowledge and skills to get hired by local health and social organizations such as the VDS partners. The Consulate staff believes that their courses are especially attractive to them because they can obtain a diploma, recognized by other organizations, that certifies specific trainings.

### Implementation outcomes

#### Acceptability

Users valued the services they received from the VDS – “a positive experience” and an “excellent resource”– especially in terms of perceived quality of health information received, listening to others’ experiences, and the availability of services. Users highlighted how the VDS staff “always helped me and answered all my questions” and “It helped me realize I had more benefits than I imagined.” Another participant stressed that the VDS helped her “make decisions and shake-out the fear and anxiety of all the external information.” Likewise, some praised the use of technology to open their services during lockdowns. On the VDS courses some users mentioned their explanations were “clear” or “easy to understand” and several expressed they wished the VDS offer even more information for them. They also valued the network of organizations, as one user said, “if they don’t offer the service, or don’t have enough capacity, they send you to those who can, to organizations working with them, like for mental health sessions.” Partner organizations complement their views with positive remarks about the pertinence of the VDS services. They highlighted their “leadership”, “commitment”, “openness”, “efficiency”, and “flexibility”. Moreover, as expressed above, VDS coordinators underscored the extent that their services addressed emergent needs of their users.

Few users expressed negative opinions on some aspects of the VDS services. Two users complained about the lack of or insufficient follow-up: “I asked for something for my son, and they told me they would get back to me, but they never did”. One user mentioned she wished the workshops had better prepared speakers and more varied topics while another one felt mistreated and preferred a two-hour drive to a different VDS.

#### Adoption

Two processes favoring the adoption of the new VDS services stood out in the interviews. The first one was the transition from in-person delivery of services to virtual interactions. The transition to online services, according to the VDS staff, increased their topics, depth, reach and diversified their users, especially of courses that granted diplomas. Likewise, they were able to continue offering regular services by telephone and began offering new ones (e.g., mental health counseling). An important facilitator was the use of digital communication platforms. The VDS increased the use of emails and social media to inform on general health topics and the new services being offered over the phone. One Consul even started giving daily briefs on social media and it soon became a good practice to inform and keep the community engaged. Moreover, the constant communication with the local Mexican community gave the VDS direct and immediate knowledge about their pressing needs, which in turn guided their services.

The second process was the interconnection with partner organizations. A pre-pandemic implementation barrier that they had already identified was verification of referral completion. During the pandemic, when communication between organizations intensified, they were able to confirm if the user reached out to the partner organization. These connections led to improved patient follow-up and user feedback by the VDS and the partner organizations, which was especially important among the uninsured users. One organization points out that “we try to make sure that we’re asking our patients how was the person that you saw, so that we can keep a list that is helpful to our patients”. The VDS recognize that one strength of their service model is case management, which proved particularly relevant during the pandemic and constitutes an area of investment on further capacity.

#### Appropriateness

The fit of the new services faced two important challenges: adoption of telemedicine technology and using an equity perspective to increase their reach. Not everyone had at-home access to the internet to benefit from telemedicine or did not have the necessary skills to make an online appointment, as was initially required to get a vaccine. One organization explains how their users “didn’t have cameras or phones that could support that [telemedicine], and they didn’t know how to use it. So, oftentimes, even if we had telehealth available, it was not easy for our patients to use it.” A user confirmed this barrier when recounting how “it is not easy for us, sometimes we cannot communicate, we have the wrong number, or we get delayed for 24 or 48 hours, and we don´t know how to respond. And by the time we get the call back it is already too late to get a test”. This was particularly relevant among older adults, who had lower digital skills and internet access, but benefited the most from telemedicine given their lower mobility and increased risk of COVID-19 complications. This particular need was therefore left unaddressed at the first stages of the pandemic, when no in-person services were available.

For the second challenge the VDS staff recognized that most of their users were female and struggled to reach working-age males. In addition, the VDS offer few services targeted to indigenous populations who can’t speak or read in Spanish. The VDS staff did not speak native languages, so they are partnering with other organizations to start designing audiovisual informative materials in indigenous languages. Likewise, few services are directed to groups with disabilities, so they are also partnering with organizations that could expand services to these diverse groups. The VDS and the partner organizations were aware of all these challenges and tried to mitigate them but were also unable to implement solutions to solve them completely and in timely manner.

#### Feasibility

This implementation outcome examines the extent to which a program can be carried out in any setting, especially among disadvantaged populations. According to the VDS personnel, the key facilitators to serve their target populations in multiple contexts during regular periods *and* emergencies are Spanish-speaking practitioners with cultural competency, in a safe and trusted space, and providing free services for the Hispanic population. As one of the VDS coordinators expressed when describing access: “It was not just the myths and all that, another great barrier for taking the vaccines or getting tested is the fear of accessing these services for fear of their migratory status.” The advantages of the Consulates’ infrastructure and personnel with cultural competency benefitted the overall network of organizations. The VDS attracts a hard-to-reach population and works as an entry point for resources and services that go beyond of the Consulates capacities because they expand their offer with akin organizations and the support from the US government. A key complement of their model is the role of community health workers. They served as a bridge between the community and the organizations to inform about ways to tailor their services to different needs and bring reliable information and insights on how to access services and resources. One partner organization highlighted that “our most important program is based in community health workers, (…) and it’s our strongest bond with the communities.”, who during the pandemic were important to convey reliable information.

#### Fidelity

Within the EquIR framework fidelity examines the adherence of the disadvantaged population to the intervention and how implementation deviates from standard practice. The long pandemic meant the intervention shifted as new needs emerged precisely to adapt to their population’s needs. The VDS and partner organizations agreed on three broad periods when implementing different modalities of service delivery. At the beginning of the pandemic, they focused on disease control through COVID-19 testing, emergency health care, and providing humanitarian assistance, such as food pantries, mostly performed remotely. The second stage was characterized by the provision of protective personal equipment, mostly masks, and for an emphasis on conveying trustworthy information. Even though lockdowns continued in different degrees, the Consulates gradually returned to work in person and conducted hybrid service delivery (in person and remote). Lastly, while they continued offering information and workshops, the main activity focused on vaccination campaigns and addressing vaccination hesitancy with specific subpopulations.

The VDS and partner organizations acknowledged that they lacked detailed protocols for service provision during disasters, so they had to reinvent themselves at every stage of the pandemic. Therefore, fidelity to their previous operation was not relevant and they had to innovate to adjust to new needs. One organization explains how they had to “think outside the box” and offer services previously unrelated to their mission, even when that means “we’re now starting to track how effective those things have been, because it’s not obvious that they are.” Even though they were resilient and creative in adapting their services, they believe a clear plan and training can help leverage the lessons learned for future crises.

#### Implementation cost

The VDS coordinators did not report having additional expenses during the pandemic; as one explains: “we did not spend more than before because we moved to virtual offices, so we stopped spending on some of the in-person perks. And all workshops were free, so no, any major expense.” However, the costs involved in the pandemic response during its different stages were not estimated and donations or indirect costs were not acknowledged as such. While the Mexican government financed the Mexican Consulates awarded some “seed money”, the VDS also had partnerships with the US federal, state and local governments to receive in-kind support such as Public Health Departments that offered guidance about COVID-19 information and content for mental-health workshops, testing and vaccination in the Consulate’s facilities, amongst others. Most of the interviewed organizations also received funding and in-kind support from governments (i.e., information, COVID-19 tests, and vaccines). Some of the partner organizations grew with the pandemic and opened new locations in their cities. The additional funding and support decreased over time, and they are currently struggling to maintain the new services offered during the pandemic. None of these changes have actually been costed.

#### Coverage

The VDS and the partner organizations do not have precise estimates of the coverage of their target populations with the new service modalities implemented during the pandemic. The VDS personnel has always been aware that geographic isolation is an important barrier because Hispanic populations in rural localities face distinct hardships. During the pandemic, they kept using the mobile Consulates to increase their reach and facilitate access to their services, but they did not report increases in their geographic coverage. Nonetheless, the VDS and the partner organizations noticed an increase in the number of users during the pandemic, especially in their workshops and events. Some identified substantially more people connected to their online broadcasts; for instance, one partner organization reported over 9,000 views on a video with COVID-19 information. Likewise, traditionally smaller groups also grew; in one organization, the average number of participants was 25, but when they switched to an online modality, the groups went to 60 and 70 persons – the most successful workshop in terms of attendance was on emotional wellbeing during the pandemic.

### Sustainability

As the VDS and partner organizations are mostly back in-person, they are realizing that the ad hoc services for the pandemic will eventually disappear when the government support retreats, or when they are no longer needed. At the same time, they are understanding which service provision adaptations are likely to become regular practice. Among the adaptations that will remain is some degree of hybrid work and service delivery. Their enhanced communication by digital platforms is something they will try to maintain. Some workshops and trainings at the VDS are likely to remain in a virtual modality to increase their reach; “because we noticed this way they can connect from their job, their homes, and even their cars, some of them will not turn the camera on, but we know they are listening”. There was a consensus that a constant stream of verified and reliable information tailored for this population must become a constant strategy to fight misinformation, and the outlet for such contents will most likely be social media. They also affirmed the importance of keeping and increasing the communication, referrals, and collaboration networks between akin organizations to strengthen overall care for the communities. One user summarized how some modifications are becoming routine:

*“I first heard about it in Facebook [mental health service]. But then I used the 111 whatsapp number where all women get to ask things and then go to therapy and get information on workshops and stuff. Sometimes they give information about the VDS, it is now more about nutrition, diabetes, high pressure, cholesterol, all that”.*

The experience of adapting their model during the pandemic left some valuable lessons for service provision. Most organizations agree that telemedicine services should continue, and they could translate into the use of more digital services, such as apps, that help with case management follow-up and to reinforce trainings. Importantly, some informants underlined the need to create and consolidate their datasets with user’s information because, as they scale-up, their current systems might not work or will not be as helpful. However, they are aware that the effort to sustain all these telemedicine options can be hampered by staff shortages.

Another lesson stressed by the members of the VDS was the importance of having contingency plans for future crises to increase preparedness. However, in the face of unexpected contingencies, availability of previous and diverse networks plays a key role in disaster response. The leader of one organization states how “many collaborations that rose during the pandemic still remain because we understood there is a benefit on working together, especially when we come from different sectors, as with education and health.” These networks should not be taken for granted but need to be nurtured with constant communication and exchanges of resources and information. The network of organizations is what they consider the bedrock of a strong response in times of crisis.

## Discussion

The study appraised the VDS adaptation of a promising outreach model during the COVID-19 pandemic. Before the pandemic, VDS were a useful program to provide health information, early disease detection, and referrals to underserved Mexican immigrants in the U.S. [16]. What this study shows is that the VDS were able to continue helping their target population when their needs changed due to the pandemic. This is particularly relevant, as prior research has documented the deleterious effects that health emergencies can have on marginalized populations [3, 4, 7].

The VDS adapted to the new circumstances with three major changes. First, they expanded their previous services to offer social assistance and humanitarian support. This expansion did not entail additional staff, so it reduced its capacity to provide their regular services. Prior research on emergency response has shown that institutional design can hamper rapid responses [14]. However, the VDS were able to quickly adapt their services as they moved along a changing crisis and managed to keep offering their regular services while adding those that were most needed in different stages of the pandemic. Second, the VDS changed from in-person to online services and then continued in hybrid models. They managed to switch between modalities by combining previous characteristics with new strategies. During the pandemic, the VDS kept being trusted organizations by providing free, reliable, and culturally sensitive services in Spanish, in a safe place, and without requiring proof of migratory status. VDS adapted to social distancing requirements adopting digital platforms to safely reach distant and busy groups. However, not everyone was able to benefit in the same degree because some lacked the skills to use the technology and others the internet infrastructure. Third, the VDS adjusted and expanded their network of partners to address the new circumstances. The VDS and the partner organizations have a two-way relationship of referrals to increase the availability of services designed for the same target population. The whole network was able to restructure its services when new needs arose by adding new partners and reconfiguring the network. Likewise, a more thorough case-management and follow-up facilitated the navigation amongst organizations. Jointly, these adaptations were key for the resiliency of the VDS against challenging circumstances.

The study used the EquIR framework to identify implementation lessons to strengthen preparedness of similar organizations against future disasters. A salient result on the adoption of these services is that these adjustments allowed the VDS to offer more services and to tailor them in greater depth while increasing their reach to a more diverse audience. The new services were generally appropriate for the target population as most were able to access and use digital platforms. Nevertheless, some groups remained hard to reach, especially working-males, people from indigenous groups, and persons with disabilities. These adaptations were feasible to implement because of adequate infrastructure for service provision and trained personnel. A frequent unacknowledged element of the network of organizations is the role of community health workers, who use the VDS, but also bridge service provision with the communities and help build trust [31]. Another implementation lesson is that context adaptability was more important than fidelity to their previous services [29].

The VDS or the partner organizations lacked clear guidelines to follow during a changing emergency as the pandemic. This meant that they had to decide their adjustments based on the available information. Therefore, the flexibility to modify their services without excessive bureaucracy was key to rapidly adjust to the different stages of the pandemic – even if the implementers did not expressly underscore it. These lessons favored that the users mostly accepted these adaptations and contributed by identifying new needs, providing quick feedback, and disseminating and recommending the services in their community. These lessons matter when organizations attempted to replicate the VDS model with hard-to-reach populations.

The EquIR framework also revealed important evidence gaps for an adequate implementation. The VDS and the partner organizations had little information on the fidelity and costs of the service adaptations. They were serving more users but had scant information on the proportion of their target population who know about their services and participate in them. From an equity perspective this information is key if organizations want to understand which subgroups are unnoticedly excluded. Likewise, the VDS recognized that they received in-kind support of different organizations, especially governments that sought the help of the VDS or their partners, as in the case of vaccines. The partner organizations reported that COVID-19 funding helped scale-up their services by adding personnel, services, or intervention sites. However, they were unable to specify the overall costs of their adaptations, which is important for replication as indirect expenses were overlooked.

The study had some limitations. Face-to-face interviews might not be the most effective method to capture some implementation outcomes, as costs and coverage, and detailed quantitative designs are needed to have this valuable information. We were able to contrast the perspectives between three key actors on similar topics but additional sources of evidence, like records from the implementing organizations, could complement these findings. Another limitation was the absence of users from the NYC Consulate. Based on the similarities between the responses of the two other key actors and the homogenous experiences of VDS users in LA, we assume the experiences of NYC users could also be similar. The comparisons in the supplementary material 2 show how expressions might differ, but the overall message is similar in both cities. Additional information about users without health insurance show how their experiences differ from those with insurance. Therefore, additional information from the user’s perspective could have been useful, but we believe the accounts of the VDS coordinators, and the managers of the partner organizations have sufficient detail to adequately answer the research questions on the adaptations of the VDS. Lastly, we collected evidence on two “sanctuary” cities and these findings need to be enriched with the experiences from other cities.

The VDS remained as a small and nimble organization, culturally tailored, doing multiple tasks with many partners. The sustainability of these models remains to be seen. Some of the specific COVID-19 components of their service model are already being descaled – especially testing and vaccinations. However, the VDS recognize that the pandemic can bolster and maintain some innovations. These changes can help establish telemedicine as a standard practice in hybrid service, online workshops to increase users, and the use of social media to constantly mitigate disinformation. Likewise, sustaining the network of organizations is considered a key feature of the VDS model that should remain after the crisis. Therefore, networking summits, outreach activities, and follow-up efforts could continue to build the necessary trust to become partners and are thus a crucial part of their preparedness for future crises. A lesson from the COVID-19 pandemic is that anticipation of and resilience to major shocks needs to be a priority for health systems embedded in highly unequal societies. Alternative public health interventions may prove essential when specific populations are systematically excluded.

Some general recommendations that emerge from this research are: (i) the need to establish general guidelines for future emergency responses in the VDS, (ii) the VDS personnel, as well as partner organizations, might benefit from formal training on emergency response as they serve a particularly marginalized populations, (iii) the VDS need to invest in social media and digital platforms to transit to viable and sustained hybrid models, in addition, personnel in the VDS might need support in the dissemination of health related information particularly tailored to their audience, (iv) the relationships between the VDS and the partner organizations should continue to be fostered as it was a fundamental mechanisms during the pandemic. In addition, there might be learnt mechanisms to improve their synergies such as follow-up of referrals, (v) the VDS need to improve their reach to serve Mexican immigrant males, indigenous, and population with disabilities, and (vi) the VDS should place more attention to costing their tasks, as this may help to secure adequate financial resources, in this respect, it is fundamental to underline that the Mexican government should continue to support the VDS as it is a relevant service to Mexican migrants, and particularly to those who are undocumented.

## Conclusion

The VDS is a flexible and trusted public health intervention that can be strengthened to improve health outcomes of a vulnerable population. The study documented how the VDS in Los Angeles and New York City responded to the COVID-19 pandemic to keep providing outreach services to Mexican migrants living in the US. It also shows that during the pandemic, the VDS and partner organizations configured a network that innovated in outreach and service delivery strategies to users. The VDS are a relevant example of how to implement interventions with an equity perspective that complement and reinforce the overall health system because they can reach and tend to groups that standard practices cannot. Universal health policies like COVID-19 testing and vaccination require tailored services for the needs of immigrant populations to expand its coverage and increase its effectiveness. The VDS also illustrate a way to tackle social determinants of health –i.e., unemployment, housing insatiability, and food security– with a bottom-up, flexible, context-specific, and people-centered public-health intervention. All VDS tend to Hispanic populations of primarily Mexican immigrants and thus have the potential of becoming the blueprint of a relevant and equitable service model to consulates in the US from other countries, whose populations face similar challenges, and to organizations providing services to hard-to-reach groups with specific needs. Its main features can be replicated in Consulates in other states and in organizations targeting the same population, while they also help explain how the VDS remained relevant during the pandemic despite their small size. Overall, the results of the study show the potential of the VDS to contribute to disaster preparedness and mitigation, strengthening the health system resilience during times of crisis.

## Electronic supplementary material

Below is the link to the electronic supplementary material.

Supplementary Material 1. Interview guides

Supplementary Material 2. Table SM1. Selected quotes on implementation outcomes by city and informant

## Data Availability

The datasets generated and/or analyzed during the current study are not publicly available due to the confidentiality agreement with participants but are available from the corresponding author on reasonable request.
