# Supplementary material for: Adaptation of a community health outreach model during the COVID-19 pandemic: the case of the Mexican consulates in the United States of America
Source: Int J Equity Health. 2023 Jul 25;22:138. doi: 10.1186/s12939-023-01911-9 (PMC10369756; doi:10.1186/s12939-023-01911-9)
Supplement: Supplementary file 1 — Supplementary Material 1. Interview guides [file 12939_2023_1911_MOESM1_ESM.docx]

**Supplementary Material 1**

**Interview Guide for Ventanillas de Salud (VDS) personnel**

Study Questions: Oral Consent

Thank you very much for agreeing to participate in this study. We will begin with the interview, which should not take more than an hour. I will keep track of the time.

1. I understand that **before the COVID-19 pandemic**, the main functions of the VDS were to provide information and advice on healthy lifestyles, perform immunizations and early disease detection, and refer patients to community clinics. We know that the pandemic changed the usual way of working and that these modifications varied according to the stage of the pandemic.

- Could you tell me which were **the main services** that the VDS added during the pandemic?
- Of those services, which ones were more important at the beginning of the pandemic, in contrast to more recent months? And which ones became increasingly important?
- Do you think there **was any service that was not appropriate** for your target population, for example, because they had different needs than expected? Which one(s)?
- Was there any health or socioeconomic need in the target population that the VDS could have addressed but, for one reason or another, remained pending? Which one(s)?

1. It's impressive all that you had to do to address this pandemic. What do you consider to be your **organization's greatest achievements of this new VDS strategy**?

- And what do you think were/are **the biggest obstacles** they faced?
- Could you mention an **example** of how you addressed that obstacle?
- In your opinion, which of these services had the **greatest acceptance** among its users during the pandemic? What elements could explain it?
- And which of them had **less acceptance**? What elements could explain it?
- Was there any group among the population served by the VDS that, due to their characteristics, required more attention or **special attention** due to their characteristics? (If clarification is needed: For example, people who only speak Spanish, people with essential jobs, or people with pre-existing illnesses).
- Similarly, did you recognize if there was **any group that preferred not to take advantage of the resources** of the VDS?

1. Now I would like to ask you about some of the details of how this new strategy was implemented.

- What were the **main reluctances** for the organizational change of the VDS during the pandemic?
- Did it require **additional personnel** compared to before the pandemic? In addition to human resources, do you remember any extraordinary but important expenses that the VDS had to incur during the emergency?
- Is there any **resource that you would have liked to have** and that you consider necessary to have for future emergencies?
- In general terms, **to what extent do you consider that they were able to implement the VDS strategy as planned**? Put another way, what adjustments did they have to make on the fly because the conditions were not as anticipated?
- To what extent do you **think the new services will be maintained** as the pandemic ends? What elements do you consider essential to sustain the new VDS services over time?

1. If you had to **recommend** **to other VDS** how to address an emergency like this, what would be the **central implementation elements** that you would highlight?

Thank you very much for your time. Your answers are very valuable and help us a lot to achieve the study's objectives.

**Interview Guide for Partner Organizations**

Study Questions: Oral Consent

1. Can you tell me about the **original objectives of your organization**?

- **Before the pandemic**, what were your organization's main services?
- Which **populations** are you directed to?
- What were the biggest adjustments you made when the **pandemic started**?
- Did you continue to adjust as the pandemic progressed? Did your target population change?
- How has your cooperation been **with the VDS at the Mexican Consulate** during the pandemic? What are the mechanisms or processes of collaboration?
- What would you say were the **main needs of Mexican residents** during the pandemic?

1. It's impressive all that you had to do to address this pandemic. What do you consider to be your **organization's biggest accomplishments during the pandemic**?

- And what do you think were your organization's **biggest obstacles**?
- Could you mention an **example** of how you addressed that obstacle?
- In your opinion, which of these services had the **greatest acceptance** among your users during the pandemic? What elements could explain it?
- And which of them had the **least acceptance**? What elements could explain it?
- Was there any group among the population you serve that required more attention or **special attention** due to their characteristics? (If clarification is needed: For example, people who only speak Spanish, people with essential jobs, or people with pre-existing illnesses).
- Similarly, did you recognize if there was **any group that preferred not to take advantage of the resources** offered by your organization?

1. Now I would like to ask you about some of the details of how you implemented your strategy during the pandemic.

- Did you require **additional personnel** from what you had before the pandemic? In addition to human resources, do you remember any extraordinary but important expense that you had to make during the emergency?
- Is there any **resource that you would have liked to have** and that you consider necessary to have for future emergencies?
- In general terms, **to what extent do you consider that you were able to implement the strategy as planned**? In other words, what adjustments did you have to make on the fly because conditions were not as anticipated?
- To what extent do you **think the new services will be maintained** as the pandemic ends? What elements do you consider essential to sustain these new services over time?

1. If you had to **recommend** **to other organizations** how to address an emergency like this, what would be the **central implementation elements** that you would highlight?

Thank you very much for your time. Your answers are very valuable and help us a lot to fulfill the study's objectives.

**Interview Guide for users of Ventanillas de Salud (VDS)**

Study Questions: Oral Consent

Thank you very much for agreeing to participate in this study. We will begin with the interview, which should not take more than an hour. I will keep track of the time.

- Could you tell me what led you to contact the VDS during the COVID-19 pandemic?
- How did you find out about the VDS services?
- During those events, what topics did you need the most help with?
- Which VDS services did you use?
- What did you expect the VDS to be able to help you with?
- What issues were left unresolved for you?
- How would you rate your experience with the VDS?
- Would you use the VDS services again? What is the reason?
- What would you recommend to the VDS to function better?
- On the recommendation of the VDS, was it necessary to go to another organization? What does this organization do? (If you went to several organizations, ask about each one separately)
- How would you rate your experience with the other organization?
- What issues were left unresolved for you?
- Would you use the organization's services again? What is the reason?
- What would you recommend to the organization to function better?
- In addition to the care, you received at the VDS and the organization, did you or your family have to go to a health center or hospital?
- Can you briefly describe your experience?
- Did the recommendations from the VDS and the organization help you?
- Finally, let me ask you some last questions about yourself:
  1. What is your age?
  2. What is your highest level of education?
  3. How many years have you been living in the United States?
  4. Do you have any chronic illnesses such as diabetes or hypertension?
  5. Do you have health insurance in the United States?

Thank you very much for your time. Your answers are very valuable and help us a lot to achieve the study's objectives.
