# Supplementary material for: Adaptation of a community health outreach model during the COVID-19 pandemic: the case of the Mexican consulates in the United States of America
Source: Int J Equity Health. 2023 Jul 25;22:138. doi: 10.1186/s12939-023-01911-9 (PMC10369756; doi:10.1186/s12939-023-01911-9)
Supplement: Supplementary file 2 — Supplementary Material 2. Table SM1. Selected quotes on implementation outcomes by city and informant [file 12939_2023_1911_MOESM2_ESM.docx]

**Supplementary Material 2**

Table SM1. Selected quotes on implementation outcomes by city and informant

| Theme | New York City | Los Angeles |
| --- | --- | --- |
| **Acceptability**  Perception of stakeholders in implementation | VDS: “These new services, like the food pantries, were very necessary for our community, for securing health and provide food security”. “Some people did not understand why they needed a vaccine. The consulate became a place where they could receive the information in their own language, find community leaders who were Hispanics looked and talked like us, all that built trust”.  ORG: “It was helpful to us because we had patients that appreciated their service [VDS]. I think that their outdoor pantries were very necessary for our community, to have that security of our health.”. “But our regular patients who are uninsured and undocumented, they know us. So they would come and be like, hey, are you doing this? Yes. And then they would tell everybody, all their friends and everything about coming to us because they knew it was safe”. | VDS: “Generally, participants are motivated. We give them a certificate of participation for the workshops, which they take, and they stay motivated and want to keep learning, so they keep connecting into the workshops.”  ORG: “Express how grateful we are to the Los Angeles window for their leadership and for the flexibility they have had in collaborating with us. And knowing that there is such a professional team behind trying to push to improve the health windows (…) It is very gratifying to see how serious and professional this service is, which is nothing more than filling in the holes”.  USERS: “I had a very positive experience. I´ve learnt a lot and they helped me a lot, my mom as well”. “It helped we understand what was going on. They gave me good information”. “I´m thankful that they answered all my questions”. “They presented excellent resources. I enjoyed the ones on health service access, how to handle it when you are uninsured, and even when you have one.” “They trust in telling us what they really need. In the workshops the ladies are not afraid of telling me, hey, we need to know about this or that.” |
| **Adoption.**  The intention or utilization of equity elements into implementation. | VDS: “She helps people get the appointment at the hospital. She takes the case from the beginning until the end, she stays with them not only for the appointment but all the way. We finish when the persons has enough information to make their decisions.”  ORG: “Well, the health windows are here to help us. And if they do, that is, if they have that capacity, they do it. And if they can't, they refer you to people who can help you, that is, they tell you we don't have this resource here, but we have these associations that are with us from the State or whatever, and they can help you to have a therapy or those things you need. So, it's important. They are always there to support you or help you with what you need”. “The issue of networks is another lesson. The network becomes even more vital in the face of a pandemic. I mean, who's going to do the vaccinations? A health department, right? Who is going to give the financial support? Well, maybe an organization dedicated to that. Food pantries, community organizations have the connection to the community and churches. So, as part of the work or achievement that the window is doing is to help people enroll in the public hospital system”. “We transitioned to telemedicine quite fast, we knew we had to keep providing services, and right away”. | VDS: “We redirected all calls so we could address all health topics, one-on-one calls where they asked us how to get tested, what happened with people travelling, where could they receive health services, or shelter at home, if they had to be separated from their family when someone was infected”. “We have weekly workshops and sometimes three times a week. And I take the opportunity to publicize the other workshops, especially the ones on mental health, and that same community helps us spread the word, and that is how people come and keep benefitting from them”.  ORG: “Our mutual partnership has worked very well, that´s the truth. We give them the content for the workshops, resources, and help them identify the persons that may use these services. The promoter working with the Consulate is very efficient, she knows their services, and she helps them decide where to send them, she reviews the case and helps them assess the information”.  USERS: “I always wished for this type of updates, access to this technology, because oftentimes as parents we need to understand that a mental health issue does not equal to being crazy. So it was excellent to have these conversations [mental health services]”. “I will definitely use it again [VDS]. They called me by phone, and I knew they cared about me. (…) And even if they only give you a flyer, they are giving you information and that is like a door that opens to the place you need to arrive, it has the information you need”. |
| **Appropriateness** Relevance or perceived fit of the implementation in the disadvantaged population. | VDS: “More complicated for people, for example, the elderly when you have access to technology, when you know, when you have email just like that. I already got the link. Thank you so much. See you tomorrow for my date. But the two older adults obviously come with this technology barrier”. “There was an explosion on telemedicine so people could speak to a doctor, but our population was limited by their access, not only by language, but for the internet connection, technical end economic challenges. You need a good internet connection, and you need knowledge”.  ORG: “We did convert to a lot of telehealth, but the problem with our population typically is that is either a homeless or low-income”. “A lot factors in our population meant they couldn't connect, they didn't have phones, they didn't understand how to get on telehealth. They didn't have cameras or phones that could support that, and they didn't know how to use it. So a lot of the times, even if we had telehealth available, it was not easy for our patients to use.” | VDS: “A lot of people didn´t want to take the vaccines because they thought it would affect their future migration status. (…) The same with food stamps. (…) Our community was really scared”. “We know that our community of older adults still has a lot of obstacles with the technology and that happened a lot during the pandemic”.  ORG: “We contacted them in 2021 because I was interested in creating flyers in indigenous languages to be able to tell them that we had vaccines, that we had workshops and so on. So, I think that for this group, we still have a long way to go”. “If you're older, especially, the challenges were difficult because you didn't know how to use a phone effectively like Zoom, essentially, and you couldn't really speak the English enough to navigate some of the windows.”  USER: “It was very useful being connected by telephone, and receiving information, and putting us on the [service waiting] lists and making the appointments for us. That was perfect because sometimes that is not for us, it is not easy for us, sometimes we cannot communicate, we have the wrong number, or we get delayed for 24 or 48 hours, and we don´t know how to respond. And by the time we get the call back it is already too late to get a test”. |
| **Feasibility**  Extent to which a program can be carried out in any setting, especially among disadvantaged populations. | VDS: “We are very focused on NYC. You need to have trained staff in helping people navigate the health system. Because we have some programs in NYC but not in Connecticut; they have different programs, different resources. You need to explain if they are eligible, the benefits, how to connect them with other services, especially according with their migration status. One of the new services was COVID, information, vaccine access, all that”.  ORG: “We focus on the people without resources, migrants who are uninsured or cannot pay. Sometimes they do want to pay but it is too expensive. So, according to their income, we offer reduced costs”. “There were essential workers, which became sort of a broad category. It was not just health care, but it was like bodega workers or like restaurants initially because they had to stay open. And a lot of our patients who are undocumented and Spanish speaking were working in these kinds of fields.” | VDS: “It was not just the myths and all that, another great barrier for taking the vaccines or getting tested is the fear of accessing these services for fear of their migratory status”. “All our people giving workshops present in Spanish, even if it’s their second language”.  ORG: “The health promoters did not just put into practice what they learned in terms of the list and how to access misinformation. But the other time, most of them greeted them and informed them by sharing their experiences. They were the ones who were working to give people information and then eventually giving information about the vaccine.”. “Our most important program is based in community health workers, which has lasted for more than 10 years and it’s our strongest bond with the communities. We recruit community leaders (…) and they work in the streets, in the schools, local governments, and of course with organizations such as the Consulate”.  USER: “They [VDS] sometimes offer health fairs, (…), I saw them offering community resources, like the health services. There they explained these things to us”. |
| **Fidelity.**  Adherence of disadvantaged population to the intervention. | VDS: “One of the biggest challenges was to respond to the most urgent needs of the people. (…) who was dying, how should we communicate, the role of the Consul”. “The issue with food pantries completely revolutionized. We already knew about the need, but the pandemic struck so hard, that a lot more people started being considered as food insecure”. “We had to come up with COVID-19 tests, which weren't available at the time and we got them relatively quickly. We had access in late March and early April 2020 and then we also procured vaccines relatively quickly to get the public vaccinated. Now we are going to be implementing this Friday program that is like this in the event of an accident, in the event of a catastrophe or something like that, how can we be prepared for it to be something that we are also going to start implementing as part of the same efforts that we are doing.”  ORG: “Do we go back to the plan? Should we start tweaking the plan? So this is something that we bring up in the safety meeting. Constantly tweak your plan”. “It was around the third month of the pandemic when we realized we didn´t have to shut down and forbid everything. We decided to reopen with multiple measures, registering who went in and out, the temperature, washing hands, masking, and we went on”. | VDS “Our services changed, and we had to leave behind preventive services, me left diabetes, cholesterol, chronic disease, what we normally do. Everything focused on Covid, in how to support the community who lost a lot, financial security and food insecurity”. “Something that happened in all Windows, not only in LA, is that we had to get creative, not really on the outreach, but being creative on the services that we had to bring to the community”.  ORG: “Even though there was a lull when we had the shutdown, I think the way we were able to continue to provide services to the community was very efficient. Yes, in a different way. But we continue. We keep doing it. It is felt that it is a welcoming environment, that it is a safe place and that is what our Latino or Spanish-speaking doctors, or our staff, who are bilingual or who are different from other clinics.” |
| **Implementation cost**  Costs involved in the implementation of equity-focused elements | VDS: “The federal government was giving money for COVID-19. So we always make sure that we try to cover access to our populations when we get federal money to try to make sure everyone has equal access”, “[Economic support] It is going to vary by locality, depending on where the VDS is located. In here we received a lot of support from the City, especially with testing and vaccines”.  ORG: “I don’t know exactly if our budget increased because of the additional services. We had been growing exponentially in the last 5 years, but I wouldn´t be able to say how much was due to the pandemic”, “I think we got COVID money, and I think it covered a lot of things. So initially it was very hard to get things.[…] Believe it or not, we stayed afloat. Everybody worked from home and those medical providers who volunteered to come in, they came in. We did not really have to expand the staff because 99% of them had to work from home. And our medical service, you know, we just kept that afloat so we didn't have to expand the services." | VDS: “Contact was made with the Department of Public Health. That relationship already existed, but the Department of Public Health has different departments. We finally managed to find someone who could support us and little by little we built a relationship.”  ORG: “My organization changed a lot, the whole program. We expanded and restructured. (…) Before the pandemic we didn´t work as close to our clients, now we are closer to them”. |
| **Coverage.**  Degree of reach, access, and coverage of the intervention among the disadvantaged population | VDS: “We serve more people now. That generated an opportunity to expand the access to telemedicine in a way that we can cover the most affected groups. The same happened with public hospitals.”  ORG: “Whatever the videos, I think they went to more than 9,000 thousand views. It was important for the institution, because that way we already know that is there and that if they see it in the end, it is the same name, it is the same number and it is the same email where they have to contact me to get these services”. | VDS “I believe that one of the achievements that we had was in education. We only managed to reach people who came here to the consulate and time is so limited that it was very difficult for us to give them information to put into practice later. But since the pandemic, since there was no other way, we had to start these virtual workshops and with various organizations (…) There were a lot of people, especially since being online you could connect with people from other places who don't necessarily go to the consulate. So we had a lot of people. The first was a series that we gave to almost 70 people”. “An achievement for us is to have 4,000 followers in our social networks. Still, LA is very big and a lot of people do not realize that the Consulate offers much more than passports and visas”.  USER: “We saw everything on Facebook. They appeared in there or advertised their workshops. You can even see the pages from other VDS and see what they offer. I sought LA but because of the algorithm, I don´t know, I ended up watching San Bernardino” |
| **Sustainability**  Maintenance, continuation, or durability of the program through short, medium, and long-term strategies | VDS: [What will remain is] communication with community, making it virtual, getting used to providing telephone services, where they may be able to talk to a doctor, telemedicine, but also educational workshops on health topics, not only in-person, as before, but also in Facebook Live, or also closed and private groups, as support sessions”.  ORG: “Have a disaster plan. We're not right now in the time of a pandemic. While the new one is coming now with monkeypox have, start your planning now. Have a sound disaster plan in place to have your phone tree together”, “[we need] this place where everyone, all the community-based organizations or organizations that help out the community can just quickly, like with a press of a button, connect with each other.”, “We definitely grew up. Yes, we had more need of certain things. I mean, now telemedicine is part of our regular practice. I would say we have a lot more staff members. And I think we still have dedicated COVID providers to this day because we have vaccinations and tests still separate from our regular visits. So we definitely grew as an organization.” | VDS: “I didn´t know how was I supposed to help the community, I did not have a plan, so now I realize we need one (…) a plan with our strategic partners so in any given moment we can unite and help each other in whatever is necessary”. “We realized that virtual was easier for the community because we noticed this way they can connect from their job, their homes, and even their cars, some of them will not turn the camera on, but we know they are listening”, “We definitely need to keep using the technology a lot, virtual workshops, Zoom or Facebook Live, those are the thing we need to try to maintain. We are almost back to normal with our services so can now focus on increasing our reach with the community, now using email and word of mouth”.  ORG: “Lots of collaborations created during the pandemic continue because we saw the benefit of working together from different sectors, say education with us in health”, “I think that every day we are going to start getting more people back to the office. But I think there will be this change. There have been substantial changes. So. I can't say for sure that we're going to go back to 100%, but I think this is going to tighten up a bit more.”  USER: “I first heard about it in Facebook [mental health service]. But then I used the 111 whatsapp number where all women get to ask things and then go to therapy and get information on workshops and stuff. Sometimes they give information about the VDS, it is now more about nutrition, diabetes, high pressure, cholesterol, all that”. |
|  |  |  |

Informants can be identified by their acronym. *VDS*: “Ventanilla de Salud” or Health Window Coordinator in the Mexican Consulate; *ORG*: key informant from a partner organization; *USER*: a VDS user during the pandemic who agreed to participate in the study with a one-hour zoom interview.
